# The impact of family support and organization on adolescents during school closure under Covid-19 lockdown regulations in an area of South Africa

**DOI:** 10.1371/journal.pone.0288501

**Published:** 2023-08-10

**Authors:** Jane D. Kvalsvig, Myra Taylor, Kathryn G. Watt, Chris Desmond

**Affiliations:** 1 Discipline of Public Health Medicine, School of Nursing and Public Health, University of KwaZulu-Natal, Durban, South Africa; 2 Centre for Rural Health School of Nursing and Public Health, University of KwaZulu-Natal, Durban, South Africa; Group for Technical Assistance / Asian College for Advance Studies, Purbanchal University, NEPAL

## Abstract

The Covid-19 pandemic and resultant disruptions to schooling presented significant challenges for many families. Well organised families have been shown to have a protective effect on adolescent wellbeing in periods of shock. At the onset of the Covid-19 pandemic, Asenze, a population-based cohort study, was conducting a third wave of data collection in peri-urban South Africa, examining risk and protective factors during adolescence. By March 2020, n = 272 adolescents and their caregivers (n = 241) in the cohort had been assessed when in-person data collection was halted by lockdown measures countrywide. During this cessation we undertook a brief telephonic qualitative sub-study to explore whether families enrolled in the cohort were able to cohabit cohesively and undertake distance learning during lockdown. A purposeful sample of 20 families (caregivers n = 20, adolescents n = 24) recently assessed in the Wave 3 of the main study, participated in semi-structured interviews. Quantitative data from Waves 1–3 of the main study was used to measure family function, adolescent cognitive function, and profile adolescent and caregivers. The quantitative and qualitative data were integrated to illustrate the dynamics of the participants’ lives before and during lockdown. We found that families classified as well-organized before lockdown, were more likely to report co-operation during lockdown. Adolescents who were self-motivated, had access to smartphones or the internet, and were supported by both family and educators, were well-placed to continue their education without much disruption. However, few schools instituted distance learning. Of the adolescents who were not assisted- some studied on their own or with peers, but others did no schoolwork, hindered by a lack of digital connectivity, and poor service delivery. The experience of adolescence and caregivers in the Asenze Cohort during lockdown highlight the importance of family functioning for adolescent wellbeing in crisis, as well as the need for access to health, mental health, and social services, communication upgrades, and enhancements to the education system during peaceful times, to make a difference to young lives in times of crisis.

## Introduction

In March 2020, to curb the spread of the Covid-19 virus, the South African government imposed strict lockdown regulations countrywide- including school closure, limited business operations and interprovincial travel, a ban on alcohol and cigarette sales, and a nightly curfew. In compliance with lockdown regulations, The Asenze Study of Risk and Protection in Adolescence, the third wave of a population-based cohort study in the Valley of a Thousand Hills in the Province of in KwaZulu-Natal ceased primary data collection. During this lockdown period, we instituted a telephonic qualitative sub-study with selected cohort caregivers and their wards, in order to gauge the effects of the Covid-19 lockdown and the impact of school closure on families within the cohort as it was happening. This sub-study was conceptualized and conducted during the height of the Covid-19 pandemic in South Africa, when the impact of Covid-19 on the over 10 million youth aged 15–24 living in South Africa was not yet known. Young people in KwaZulu-Natal face a myriad of challenges. Service delivery such as proper sanitation is limited for many, food insecurity is widespread, and Common Mental Disorders among adolescents are prevalent [1, 2]. All of which were arguably exacerbated by the pandemic and associated lockdowns which worsened social vulnerabilities and the structural drivers of health inequities [3], as well as negatively impacting mental health globally [4]. In many developing countries the education system was not equipped to support distance learning during pandemic including lack of network infrastructures, computers, and internet access [5].

Family functioning influences the well-being of adolescents both through positive interactions such as caregiver supervision and monitoring and modelling of emotional responses; and negative interactions such as violence and abuse [6]. In times of social disruption/shocks, such as during the pandemic when many adolescents have been cut off from the social support of their schools, peers, and communities, positive family functioning can act as a buffer to the associated risks [7, 8]. Yet in such adverse events family functioning itself can be compromised, as caregivers face a cascade of additional stressors and challenges to their own mental health [9].

This paper uses a mixed-methods approach, based on data from the Covid-19 qualitative sub-study, which is interpreted by incorporating quantitative data on the participants from Waves 1–3 of the Asenze cohort study. The qualitative methods to allowed participants to explain their own circumstances and perspectives during the sudden and very strict first lockdown [10], revealing a diversity of views on the phenomenon, allowing us to identify social patterns in the responses [11]. The interpretation of these patterns was enhanced by extracting quantitative data from assessments on the family functioning, health and development of the participants conducted over a period of ten years in the main Asenze cohort study.

This paper has three central hypotheses: firstly, we surmised that a pre-existing order in the home and co-operation in daily activities would help to maintain cordial interactions between family members closeted together. Secondly, we hypothesized that if the schools-maintained contact with their learners during school closure, supporting their academic progress towards the critical matriculation examination, the learners would be more engaged in their academic careers. Cumulatively, we inferred that support from both school and family would help adolescents ride out the delays and frustrations of the lockdown experience.

## Materials and methods

### The Asenze cohort study

The Asenze Cohort study commenced in 2008 with a focus on child neurodevelopment, cognitive function, child behavior problems, and physical and mental health in the Valley of a Thousand Hills. The study area covers five adjacent local authority areas 45km northwest of Durban, ranging from peri-urban to deep rural terrain in the KwaZulu-Natal province of South Africa. The province is situated on the east coast of South Africa, and had an estimated 11.5 million people, 19.1% of the South African population [12]. In 2017 40.2% of households in KwaZulu-Natal were lower income earners, in 2018 the province had the highest number of social grant beneficiaries in the country [13].

All children between the ages of 4 and 6 and their primary caregivers living in The Valley of a Thousand Hills were eligible to participate in the cohort. A door-to-door field survey visiting each household in the area identified 2049 eligible children and their primary caregivers. Upon completion, 1581 children (88.5%) and 1437 primary caregivers were assessed in Wave 1. Wave 2 took place in 2010–2012, when the children were aged between 6 and 8, 1409 children (89.1%) and 1273 primary caregivers were assessed. Wave 3 began in 2019, with a focus on risk and protective factors in adolescence. By March 2020, when lockdown began 270 adolescents and their caregivers had been assessed in Wave 3. In December 2021 Wave 3 completed the assessment of 1176 adolescents and 1066 primary caregivers.

The data collected in each wave of the Asenze Study included information on demographics, child cognitive development, child health, child behavioural functioning and achievement, family functioning and environment, caregiver functioning and psychosocial measures.

### The Covid-19 qualitative sub-study

The Wave 3 qualitative sub-study was conceptualised and implemented in response to the Covid-19 pandemic lockdown and resultant cessation of data collection in the main cohort study.

Participating caregivers and adolescents were purposefully selected from cohort members who had already completed their Wave 3 assessments prior to the onset of the pandemic, with attention to representing all three local authority areas in The Valley of a Thousand Hills where participants had been recently interviewed, to reflect the differences in local governance, infrastructure, and resources.

The primary participants were the caregivers; the purpose and procedures of the sub-study were explained to them, and the fact that the interview was to be recorded; the interviewer asked their permission for the adolescents to be interviewed as well. There were no refusals. Following the caregiver interview, the interviewer spoke to the adolescent and once assent has been obtained, the interview proceeded.

The caregiver interviews addressed two broad themes: the interviews included questions on (a) whether, since adolescents often turn away from their parents’ guidance and look to peer behaviour, this could be causing inter-generational tensions under lockdown; and (b) how adolescents perceived their current situation because of the Covid 19 pandemic. The adolescents’ interview schedule followed similar themes with the addition of more questions on what the closure of the schools meant to them. It was sometimes necessary to contact the families once more to clarify information from the interviews.

Experienced bilingual interviewers with Zulu as their primary language undertook in-depth qualitative interviews telephonically from a convenience sample of caregivers (n = 20), and, with their permission, the adolescents (boys n = 11, and girls n = 13) for whom they cared. The interviews were conducted in Zulu using an open-ended interview schedule and digitally recorded after the respondent had given consent. It was explained to the participants that they were being interviewed so that we could understand how they were being affected by the COVID-19 epidemic and lockdown. The recordings were transcribed, and the data was anonymised using project ID numbers which linked the participants anonymously to the main study’s quantitative data collected over the three prior waves.

#### Measures taken from the main cohort study

To gain insight into the antecedents of the lockdown experiences of the twenty families interviewed in the qualitative sub-study, we extracted information on family function, child cognitive function, and education related quantitative measures from prior waves of the main study. For each of the scales selected we noted where participants in the Qualitative sub-study scored above or below the average of the 270 adolescent participants assessed prior to lockdown in the main study.

#### Confusion, Hubbub and Order Scale (CHAOS)

The CHAOS measure of household function and disorder in the family [14], had been conducted in all three waves of the cohort study. The 12-item scale included items about crowding and high noise levels and was particularly relevant to lockdown conditions. The caregiver had been asked about the way in which the household had been ordered and a score at the upper end of the scale indicated a disrupted household where there was little co-operation between family members and effective planning of family activities. Using the small sample of participants in the sub-study the Wave 1 and Wave 2 CHAOS scores were significantly correlated (Kendall’s Tau = .438, p = .032), as were Wave 2 and Wave 3 (Kendall’s Tau = .690, p = .001), giving us a consistent evaluation of order in the households of the families we interviewed. This measure was selected as we hypothesized that families who were well-organised and co-operative in each Wave (evidenced by low CHAOS scores over time) were more likely to report in the interviews that they interacted with affection and co-operation under the unprecedented closeness experienced during lockdown.

#### Kaufman Assessment Battery for Children (KABC)

The children’s cognitive skills (memory, learning and planning) were assessed using subtests from the KABC II across the three waves yielding a possible 11 scores (Atlantis and Hand Movements in all three waves, Conceptual Thinking in Wave 1, Rover in Waves 2 and 3, and Atlantis Delayed and Pattern Reasoning in Wave 3) [15]. In each wave, we noted for the adolescent the number of tests in which they had scored above the average for the entire main study sample in the first two waves. and in Wave 3. the 270 who had completed the assessments before the lockdown, as a proportion of the number of tests they had completed. High scorers in the sub sample were defined as those scoring above average in 70% or more of the KABC assessments.

In the first wave of the Asenze assessments the cognitive development of the children was assessed using sub-studies of the KABC II, and higher scores were found not only to be associated with preschool attendance and nutritional status, but also with the area where the child lived [16–18]. In a path analysis of cognitive development across the first two waves nutritional status directly predicted cognitive development as measured by the KABC scores [17]. As time passed and the adolescents had greater exposure to the environment, other factors could have influenced the developmental trajectories of the adolescents in our sample. Viewing the three sets of scores there were adolescents in the sub-study who had above average scores on all or most tests throughout, some who only had above average scores at the Wave 3 assessment, and some who remained consistently below average throughout the three time periods. This was of interest in the present study because we considered the possibility that the closure of schools during lockdown would have a stronger effect on those children who were already struggling academically, a theme which led us to examine whether the participants had continued with their studies during the lockdown.

#### Adolescent profile measures

In the Wave 3 quantitative assessments of the cohort, we recorded the adolescents’ access to media and the internet. We hypothesized that these technologies and demonstration of connectivity would be linked to adolescents’ ability to participate in distance learning. Adolescents’ self-assessment of how they fitted in socially and academically at school was measured using the Psychological Sense of School Membership Scale (PSSM) [19]. The PSSM has been validated in South Africa [20]. Adolescent perseverance was measured in Wave 3 using The Short Grit Scale, a self-assessment consisting of 8 items [21]. The adolescent’s education level was measured as whether they were currently enrolled in the appropriate grade for their age.

#### Caregiver profile measures

We also took note of the caregivers’ responses in Wave 3 to the measure of Parental education aspirations for their children, where they recorded their hopes for their children’s future educational attainment. We noted the caregiver’s level of education graded as above or below high school completion.

#### Mixed methods approach

The study used data from both quantitative and quantitative sources. The primary source of data was the qualitative interviews with the caregivers and adolescents on their experiences during the lockdown. To help us interpret these data, we used the quantitative data from previous waves of data collection to provide an indication of the respondents’ history and situation relative to other members of the cohort. While from a quantitative source, these data essentially became another source of qualitative information on the family.

#### Qualitative analysis

The interviews were transcribed in Zulu and then translated into English. They were read several times and a thematic analysis was developed through an iterative process using NVivo software, to explore how the adolescents interacted with other members of their families, and the differences between the caregivers’ views of the family interactions compared with those of the adolescents. The second area subjected to this exploratory analysis was how the adolescents coped with the interruption to their schooling. The accounts of the adolescents’ experiences in these two aspects of life under lockdown regulations enabled us to group together those adolescents whose accounts were similar and to examine the antecedent data to extract themes or patterns. Ultimately the purpose was to identify factors which might promote resilience, given that these adolescents lived in resource-poor communities which were reportedly worse affected by the lockdown regulations than those living in more affluent circumstances [22]. Families were then grouped according to their responses related to our hypotheses and the mean score on the relevant quantitative measures was compared across groups. Given the sample size formal statistical tests are not possible and this comparison is qualitative in nature, providing an indication if the quantitative data broadly reflect the expected pattern.

#### Ethics

An amendment to the application to Biomedical Research Ethics Committee at the University of KwaZulu-Natal (BE609/18), and the Columbia University of New York Institutional Review Board (AAAC2559) was approved. This permitted the ASENZE team to implement the qualitative study to explore the risk and protective factors associated with the COVID-19 pandemic. Written informed consent and assent was waived.

## Results

### The role of family cohesion in coping during lockdown

Our hypothesis was that family cohesion was important for the well-being of individual family members during the Covid-19 lockdown restrictions, and particularly for the adolescents, who were now prevented from taking part in activities outside the household. Data from Waves 1 to 3 enabled us to examine which of the antecedent factors measured were associated with family cohesion under lockdown conditions.

We divided the families into two groups: those that said they got on well and enjoyed each other’s company or only had minor tensions (twelve families), and those that had more serious disagreements (eight families). For the most part our hypothesis was correct with a significantly higher mean scores on the CHAOS scale over time in the families with serious disagreements. The CHAOS sores for the eight families reporting serious disagreements during the lockdown had a mean total of 12 points over the three waves whereas the mean for the twelve families who reported satisfactory relationships was 6.4 points.

Realistically, most families had minor disagreements from time to time, particularly in response to being homebound, but these were not significantly disruptive to the families: one caregiver said, “the children were mischievous but not more than usual”, while another said the children “they are troublesome but not that bad”.

#### Happy homes reporting cohesion between family members

Overall, the question of family cohesion was of more interest to caregivers than to adolescents whose primary worries were about disruption to their schooling and isolation from friends. This was particularly the case in households where both caregivers and adolescents reported that family members were getting on well together: the caregivers typically said the children were compliant with regulations and did not have other worries apart from missing school. For this group of families, the adolescents took the cohesion and cooperation for granted, and their attention was focussed on other issues. A typical example was in one such family where the mother expressed anxiety about being unemployed and accessing food for the family. When her daughter was asked a similar question about her anxieties under lockdown with her family, she said that living at home was easy and safe from the COVID infection, but boring, especially when her phone stopped working. When asked what made her happy, she said that she had heard they were going back to school soon.

Under strict lockdown family cohesion was achieved in different ways by different families in our sample.

*Case 1*: An adolescent from one of the families who said that they were getting on well with one another during the lockdown, and whose caregiver reported a CHAOS score of 2, thought that it was because there were only four of them. She said that the hardship for the young people in the household was their worry about missing schooling and perhaps having to repeat the year.

*Case 2*: In a case where the family CHAOS score was zero, both mother and daughter agreed that they were getting on well although the daughter was finding it hard to adjust because she had to learn to cook.

*Case 3*: A mother with two sons in a family which scored 3.5 on the CHAOS, said there were small disagreements but nothing out of the ordinary. The two children did not have much to say. One son expressed boredom, but the other was looking forward to his birthday and to eating cake.

*Case 4*: One of the families reported to be thriving under lockdown conditions with adherence to regulations, plentiful food from their own garden, an income from selling the surplus, and a high degree of participation from family members. The mother achieved this by being very strict and protective with the children, she explained “for me, the children, and the older ones, they listen because they are my children, they do what I say. I can put it like that…There will be no one who will disagree with me.” The teenage daughter in this family felt comfortable with the situation, she felt the family was harmonious because, as she put it, “it’s that we were able to work together, maybe when we were being taught something we did not know”. According to our Wave 1 and Wave 2 assessments of this family, the household had always been a calm place with low scores on the CHAOS.

#### Unhappy homes reporting tensions between family members

In both happy and unhappy homes there were common intergenerational tensions. As one caregiver expressed it:

*Well*, *there are lots of arguments as I’ve mentioned before because children are not used to doing chores during the day*, *they do their schoolwork during the day*. *Because they are at home*, *one sometimes asks them to prepare beans for cooking*. *According to them they are slaving*, *and one could see that they are quite upset by that*, *and when you ask them to fetch water*, *they really feel upset*. *There is that misunderstanding between children and parents*.

The common sources of irritation for adolescents across most of the households were having to do household chores, the boredom and worry about schooling. Although some families handled it well, disorganised families, unused to co-operating, were unhappy. The situation was exacerbated by the family size and crowding, lack of amenities at home, such as books and computers, the poor service provision (water and electricity) in some homes, and extreme poverty where food was in short supply and donated food parcels were not available. In families with a history of unresolved behaviour problems and mental health issues the interviewees told of heightened distress. The level of family cohesion affected the degree to which adolescents worried about disruption to their schooling and isolation from friends that were the overriding concerns for almost all adolescents in the sample.

In the interviews, the families we classified as unhappy had specific complaints over and above the minor intergenerational grumbles:

*Case 1*: In a family with a high CHAOS score the family difficulties predated the lockdown—one daughter became pregnant as an adolescent and the other was expelled from school for persistent truancy. The caregiver complained in the interview that the second daughter was disobedient during the lockdown and would not go for COVID testing when she had flu-like symptoms.

*Case 2*: In a disturbed family (CHAOS score 10), the grandmother who was the main caregiver for the adolescent grandchildren told the interviewer that in the past there had been previous intergenerational violence. Now, during the lockdown, intergenerational arguments flared again: she reported that her daughters condemned their mother, the caregiver grandmother, for scolding the grandchildren when they misbehaved. The grandmother said: “It’ll end up killing me because I don’t like quarrels. I feel like locking myself in my room”. She heard the children laughing about her because she had not defended herself during the arguments. She called the children’s father, and he ordered the children to move to another house on the same property, but the unpleasantness persisted.

*Case 3*: In a large family which had a history of being disorganised at times (CHAOS scores of 5, 11, 5 across the waves), the interviewees had differing views on the sources of friction. The caregiver initially said that people were getting along well, but as the interview progressed, it became clear that this was not the case. The two adolescent girls were open about the dissension in the house.

***Teenage girl 1:** Because people are staying at home, and some are no longer working. They stay together and that sometimes causes violence, and they won’t get along……*

***Teenage girl 2:** It’s just that sometimes there is quarrelling, and as you know, when you are staying together for most of the time, sometimes there is yelling and others are mischievous*.

Regarding the cause of dissension, the caregiver said that in their household there was quite a lot of quarrelling and yelling at each other over food which was in short supply and some people ate a lot. As one teenage girl put it “It is difficult as we all stay together most of the time and one would want food and it was sometimes little, and one would end up not getting it.”

### Caregiver aspirations and adolescent anxieties about schooling

These were households with limited access to reading material beyond schoolbooks, but the caregivers placed high importance on educational achievement for their wards. In answer to a question from the interviewer to the adolescents on whether they had read any magazines, newspapers, or books apart from schoolbooks, while they were at home, only two of the 24 adolescents interviewed had done any additional reading. Only one mentioned using a library before the lockdown. None of the caregivers reported any formal education beyond high school, and several had left school during primary school. However, their aspirations for their children were high. When answering the “Parent’s education aspirations for their children” during the Wave 3 assessments, the caregivers anticipated that the adolescents would complete Grade 12 (the final year of high school), and most hoped that their charges would also complete post high school qualifications. The exception was a caregiver who had mentioned that her son had learning difficulties, but nevertheless she hoped that he would complete Grade 12. During the assessments prior to the lockdown most of the caregivers in the subset of families reported that their children did homework every day, with just five children who did not. Similarly, most caregivers reported that they, or another member of the household helped with homework, checked that it had been done, and reviewed it. All but two said that they attended school meetings often. Even if the caregivers were overstating their encouragement, the responses to these questions indicated that the caregivers placed importance on supporting their children’s studies.

### Distance learning support during school closure

This issue of school closure attracted more comments than anything else in the adolescent interviews. In some cases, the adolescents were bored and lonely and simply missed their friends, and the freedom of being away from the house, chatting, or playing games with schoolmates. In other cases, the anxiety was more intense, the young people were concerned about the education they were missing, they worried that they would have to repeat a year, and that it would take longer to finish school. There were some who expressed a fear of becoming infected with the Covid-19 virus when they returned to school, and perhaps dying. We examined these attitudes in the light of their cognitive capabilities up to that time: whether they were in approximately the correct grade for their age, and what their feelings were towards the school they were attending as measured by the PSSM. We were most concerned with the support for learning that the adolescents’ teachers managed to organise during the lockdown, because it has implications for possible intervention in future crises where children are unable to attend face-to-face schooling.

The twenty families were divided into groups based on whether in the adolescents’ efforts to continue their schoolwork they were assisted by family or not, and (a) school; (b) peers; (c) whether they worked entirely on their own, or (d) whether they did not continue with their schoolwork and the pattern that emerged is depicted in Table 1.

**Table 1:** Family and school support for the emotional and educational development of the adolescent.

QUANTITATIVE									QUALITATIVE		
*Adolescent*						*Caregiver*					
Learner	PSSM	Correct age for grade	TV	Cellphone	Internet	CHAOS score	Family violence	High school education	Family cohesion	School work during lockdown
17										*NONE*
18										
20										
16a										
16b										
19a										
19b										
8										*ALONE*
9										
10										
11										
12										
13										
15										
14a										
14b										
2										*FRIENDS*
7										
1										*SCHOOL*
3										
5										
6										
4a										
4b										

#### Family support and school support for schoolwork

Some schools in the area used cell phones to good advantage where the learners were in possession of smart phones. This support and interest from the teachers kept the children motivated to do well and reduced the anxiety possibly having to repeat a year.

Only six adolescents (four girls and two boys) in the sample were assisted by their schools to keep doing their schoolwork during the lockdown. Five out of the six respondents reporting school support were high scorers on the cognitive tests that had been administered during the three waves of data collection in the Asenze project. The exception was a younger boy in Grade 9 who only had above average cognitive scores in Wave 3. All of them were progressing well through the school grades: four were in the correct grade for their age indicating that they had not failed any grades, and twin girls in grade 12 were younger than the standard age for this grade, indicating that they started school early.

These learners were not only assisted by their teachers, but also worked on their own and with friends. They set goals for themselves and worried about the fact that they might have to repeat a year. Three of this group mentioned having to overcome the fear of catching the virus when they returned to school, and of bringing it home to their families.

The schools had different approaches to distance learning. In one, extensive case work was given by the mathematics teacher. One respondent worked through them with school friends who lived nearby. One of the friends was the class representative who maintained contact with the teacher on behalf of all the learners. This respondent said that the system worked well because the class representative was accustomed to being in contact with the teacher and shared all the questions and responses with the whole class. If they individually contacted the teacher, some of this extra information might have been missed.

Another learner reported that while she had been given work to do by her teacher, but there was no feedback from the teacher upon completing the work “You had to work individually and there was no-one to correct you”. She was distressed by the lack of personal contact. She said she wanted to return to school but had lost hope of passing the grade.

They had managed to keep learning because their school used WhatsApp groups to stay in touch.

Each learner who received distance learning support from their school had above average scores on the “Grit” self-assessment measure of goal-directed behaviour in Wave 3. They were mostly anxious to do well and some worried about the time they had lost, and planned to work especially hard when they got back to school. In several instances it was obvious that the caregivers were supportive of their efforts as well.

In this way some schools in the area had used cell phones to good advantage where the learners were in possession of smart phones. This support and interest from the teachers kept the children motivated to do well and reduced the anxiety about possibly having to repeat a year. The adolescents who had been helped by the school or by individual teachers had managed to keep learning through the school’s use of WhatsApp groups. They were mostly anxious to do well and some worried about the time lost, and worked especially hard when they got back to school. Clearly, they were the ones whose schooling had suffered least. All of those who were assisted by their schools during lockdown were also assisted by their families. The families had shown themselves to be well-ordered on the whole, and only two families reported minor disagreements among family members. Only one adolescent was critical of the school’s assistance to her during the lockdown: she had received work from the teacher but had had no feedback from her. Overall this group enjoyed a good relationship with their families, teachers and classmates. They were assisted by access to technology, as all had access to cell phones and TV and six out of the seven had access to an internet connection. Despite their circumstances under the strict lockdown, they had reason to be optimistic about the future.

The importance of support from both home and school, as well as technological tools in the form of phones, internet access and television were necessary in bolstering the adolescent’s determination to set goals for themselves, and to follow them. Personal contact from teachers and feedback was more stimulating than just the distribution of worksheets. These interventions helped the adolescents to have a positive outlook on the future. Those with proven cognitive competence were able to benefit from this support and maintain a positive outlook about the future despite the lockdown measure, shortage of food and separation from their friends.

#### Family support and schoolwork with friends

Two girls and a boy received no distance learning support from their school but worked with friends and on their own. The two girls were high scorers on The Short Grit Scale. One of these girls did some revision on her own and with her friend when she came around to visit. The other girl had been above average in every cognitive test over all three assessment waves. She joined a WhatsApp group organised by her peers but was unable to keep working with them when her phone malfunctioned, and she then continued working on her own. She said she had been determined to pass this grade; and now she feared that she would have to repeat it because of the school closure. The boy said he was only visited by one friend who he worked with who was also missing school and the companionship.

#### Schoolwork done alone with or without family support

There were three boys and five girls who worked alone, some of whom had family support. This was the largest group in our sample and was a truly mixed group. The boys were dismissive of the small amount of work they had been doing at home. Two of them had below average cognitive scores in the first two waves and had only done well in the last set of assessments; however, one of them had above average scores over the three waves of testing.

The five girls, on the other hand, each expressed anxieties about missing school and tried hard to work on their own but found it challenging, as one girl expressed “I don’t understand some of the things when I study alone. There’s no one to help me understand.”. All the girls had previously scored well on cognitive tests.

There were two disruptive families in this group of self-motivated learners; they both had high CHAOS scores. the two girls in the one family were capable learners with high scores on cognitive tests, and high scores on the PSSM Scale. They had phones, internet access and a TV and above average scores on Grit Scale. These girls were outspoken in their criticism of life at home but seemed confident with respect to their schooling. These girls seemed able to rise above the trials and tribulations of the lockdown and looked forward to their return to school.

The girl in the second disruptive family had access to the internet and television but had low scores on the *PSSM*, Short Grit Scale. Those who were self-motivated enough to work on their own reported the difficulty of being without anyone to guide their studies and answer their questions.

Some of the self-motivated group who studied on their own, lived in reasonably favourable circumstances and some also involved their friends in their lockdown learning. but in the absence of help from the school the adolescents battled to keep going with schoolwork.

Others battled with low cognitive competence (as evidenced by low KABC scores), little help from electronic media, or from their argumentative families. All of them could have benefitted from assistance from teachers if it could have been arranged, and more virtual contact with the outside world.

#### No schoolwork done at home

There were four boys and two girls who did no schoolwork during the lockdown. All but one were low scorers on the cognitive tests, but they did worry about missing school. Some said they did not want to get left behind, and one said that he wanted to go back because he missed his friends. The mother of a boy and a girl in this group said that although her children wanted to return to they felt safer from the COVID virus when they stayed at home.

One mother told the interviewer that her son needed face to face learning with someone who was patient with him. A girl who had been expelled when it was discovered that she visited a friend without her parents’ permission, said she now wanted to go back to school but had not yet plucked up courage to speak to her parents or the school about it. One boy, attending a school for children with learning difficulties, said that some children study online but he does not have a smartphone so it is difficult for him to understand the work like the others who are online.

Most of those who did not study at all during the lockdown needed more specialised help: perhaps parent-teacher virtual meetings to explain how caregivers could help their children; electronic media and assistance in connecting with online learning; a counsellor for the girl who had dropped out of school; and the continued functioning of the special school.

## Discussion

The Asenze cohort offered unique insight into the effects of the strict South African COVID-19 lockdown procedures on adolescents in relatively poor peri urban and rural communities. The main cohort study has extensive data on more than 1400 participants over a ten-year period, quantitatively assessing family and caregiver circumstances and measuring the health and development of the children. Building a qualitative study with links to the quantitative assessments in the main study has enabled us to make connections between the assessments prior to the lockdown, and the way in which the participants have viewed their own experiences during lockdown. In order to develop effective interventions to mitigate the impact of the lockdown regulations on the adolescents’ future lives, we need to understand the familial and community context in which these young people made decisions about their educational involvement, and the contextual constraints that limited these decisions.

At the time of the lockdown, most of the Asenze Cohort adolescents were in high school, working towards the final school leaving examinations which would determine whether they would be able to enter higher learning institutions. Overall, there was a difference in the way the girls faced the break in schooling when compared to boys. The girls expressed anxiety more often, and more of them were in the groups that had carried on with their schoolwork at home. The boys were less open about their worries and more likely to freely admit that they had done little or no schoolwork,

Ideally, all the adolescents should have been supported by their families and the school in their journey towards adulthood, but this was not always the case. When the data from both the interviews and the quantitative measures were combined it became apparent that adolescents who had adequate support demonstrated in both, were more likely to be able to ride out the storm of the pandemic in their lives.

The National Income Dynamics Study and the Coronavirus Rapid Mobile Survey reported that anxiety over return to schooling amongst South African parents was higher amongst the poorest 40% of households [23], that mental health issues negatively influenced household worry, but anxiety was significantly lower if children received a free meal at school.

Adolescent anxieties around returning to school centred on possibly having to repeat the year, contracting Covid-19 at school, and having difficulty in catching up. These were not only expressed in this study but also in other parts of the country. This was also a time in their lives when social contacts outside the family had become important to them. Both of these processes have been disrupted in an unexpected manner, and few were able to make use of the technical facilities available in more affluent communities for continuing their education and maintaining their friendships. Others have noted that the effect of the pandemic has been to deepen structural inequalities in education in South Africa [24, 25].

The way in which the families were able to function became all the more important in lockdown. This sample contained participants from the main Asenze study who had different family backgrounds, allowing for a rich and nuanced account of factors which impacted on adolescents.

Revisiting our three hypotheses, the first hypothesis suggested that a history of order and co-operation within the family would be beneficial to families when they were forced to be together during the unsettling onset of the pandemic and lockdown. In South Africa families experienced varying degrees of anxiety over the risk of the infection itself, financial hardship and food shortages, and a degree of social isolation [26]. Family disorganisation and instability as measured by high scores on the CHAOS scale were linked in another study with increased conflict between family member [27]. The interview data in the present study supported the view that lower CHAOS scores in all three prior assessments, that is, stability and order in the family, were associated with reports of good family cohesion during the strict lockdown.

The second hypothesis dealt with the way in which schools assisted the adolescents to maintain their academic progress. In a press release in July 2021 UNICEF South Africa reported that “rotational attendance, sporadic school closure and days off for specific grades resulted in children losing 54% of learning time” [28]. The report estimated that between 400,000 and 500,000 had dropped out of school, and these were probably children living in settings with household poverty, similar to the Asenze study area.

In this sample, few educators kept in touch with the learners creating an unfortunate hiatus in their academic progress. The Education Department issued a directive that all learners should proceed to the next grade at the end of the year, but this has meant that the work that was missed during lockdown and might not have been covered in what remained of 2020, could impede progress in the following year, possibly creating gaps in their understanding of the curriculum subjects. While some highly motivated and competent learners would be able to make up for lost ground, the rest would suffer [29].

The third hypothesis concerned adolescents living in under-resourced areas and suggested that adolescents who had support from both parents and teachers might be sufficiently goal-directed in their study habits and well-informed academically to regard their futures with confidence. This was the case in the five families in our group who were reportedly well-organised at home, and the adolescents who had access to phones and the internet to maintain contact with the outside world and had been given work by their teachers (see Table 1). Even many of those who were supported by a stable home environment, but not given schoolwork by their teachers were sufficiently goal-directed to work on their own. Unfortunately, there were adolescents whose cognitive scores were low, who were not given work by their educators, and/or who had serious quarrels with their caregivers, who were not doing any schoolwork of their own accord.

Analyses of the first two waves of the Asenze study have shown that early health, nutrition and stimulation were important in developing cognitive skills during the preschool period [16–18]. In the present qualitative sub-study several themes emerged with respect to resilience under the crisis in the education system. Cognitive competence had a bearing on the way in which the adolescents were able to maintain an interest in their academic work but help from the school itself was an important influence in maintaining academic work, and not just in supplying worksheets, but for teachers to give feedback and answer questions. School counsellors and remedial teachers would have been beneficial to those with specific difficulties either emotionally or academically, but these were not available. The adolescents who did not see the importance of doing schoolwork at home during the lockdown may have struggled when schools resumed [30]. The fact that more boys than girls fell into this category suggests that work needs to be done with parents to encourage and value academic competence in boys.

### Strengths and limitations of the study

As in all qualitative studies it is not possible to determine how representative of the population the issues raised by participants were. However, the anxieties expressed by the adolescents around disruption of schooling and the risk of returning to school during the pandemic have been widely reported [25, 31]. What this study adds is the ability to link the presence of family organization, support and school outreach to earlier family and child characteristics measured at average ages five, seven and fifteen. The usefulness of the telephone interviews was largely dependent on the interviewer’s ability to establish rapport through this medium: the inclusion of Asenze participants who had recently been assessed for the third wave of the main study was probably another advantage in this respect, but nevertheless sensitive issues, like an increase in violence against women in the household, were unlikely to be raised under these circumstances. However, the diversity of the families and the range of experiences, taken together, have given rise to a lively account of the major issues for families in poor communities.

## Conclusions

Among the many changes to lives in this community we have chosen to look at the support the adolescents received from within the family and from the school with a view to understanding what services were needed for families in trouble, and what additional education programmes should be offered so that in future learners can weather disruptions in their education more effectively. This account of the interviews together with the background information derived from the main study drew attention to three main issues for the adolescents and suggests priority interventions.

Firstly, pre-existing conditions in the family such as a stable and co-operative lifestyle were associated with better educational outcomes for adolescents at a time when the threats to health were alarming. Secondly, cognitive development from an early age enabled some adolescents to benefit from the limited support available, and thirdly access to technology provided the necessary tools for maintaining links with educational stimulation. The small group of adolesents who had all three of these advantages were more confident and hopeful for the future.

Adolescents in the Asenze study demonstrated that they could cope even during a major public health emergency if they had access to resources that they could draw on to sustain wellbeing. Adolescents without these resources struggled.

The Asenze cohort demonstrates the value of providing sustained health and social supports (eg, nutrition and early childhood development) to low-income communities. The study also demonstrates the need for additional resources in these communities during a public health crisis to enable all young people to thrive. The need for the tools for home-schooling was a central theme. The experiences of these young people show that it is particularly important in low income areas that affordable communication networks are available in emergency situations, and that teachers are trained in using new technologies [32]. High quality distance learning programmes in several languages could raise the level basic education provided for South African children. While the COVID pandemic has produced a flurry of activity in this direction, it has mainly assisted those in more affluent communities [22]. The actions we are proposing are important to close the gap and eliminate the wide inequities in life chances that young people currently experience.

## Supporting information

S1 Dataset (SAV)
